# Flower power in the city: Replacing roadside shrubs by wildflower meadows increases insect numbers and reduces maintenance costs

**DOI:** 10.1371/journal.pone.0234327

**Published:** 2020-06-09

**Authors:** Karsten Mody, Doris Lerch, Ann-Kathrin Müller, Nadja K. Simons, Nico Blüthgen, Matthias Harnisch

**Affiliations:** 1 Ecological Networks, Technical University of Darmstadt, Darmstadt, Germany; 2 Magistrat der Stadt Riedstadt, Riedstadt, Germany; Universidade Federal de Uberlândia, BRAZIL

## Abstract

Massive declines in insect biodiversity and biomass are reported from many regions and habitats. In urban areas, creation of native wildflower meadows is one option to support insects and reduce maintenance costs of urban green spaces. However, benefits for insect conservation may depend on previous land use, and the size and location of new wildflower meadows. We show effects of conversion of roadside plantings–from exotic shrubs into wildflower meadows–on (1) the abundance of 13 arthropod taxa–Opiliones, Araneae, Isopoda, Collembola, Orthoptera, Aphidoidea, Auchenorrhyncha, Heteroptera, Coleoptera, Nematocera, Brachycera, Apocrita, Formicidae–and (2) changes in maintenance costs. We assessed the influence of vegetation type (meadow vs. woody), meadow age, size, location (distance to city boundary), and mowing regime. We found many, but not all, arthropod taxa profiting from meadows in terms of arthropod activity abundance in pitfall traps and arthropod density in standardized suction samples. Arthropod number in meadows was 212% higher in pitfall traps and 260% higher in suction samples compared to woody vegetation. The increased arthropod number in meadows was independent of the size and isolation of green spaces for most taxa. However, mowing regime strongly affected several arthropod taxa, with an increase of 63% of total arthropod density in unmown compared to mown meadow spots. Costs of green space maintenance were fivefold lower for meadows than for woody vegetation. Our study shows that (1) many different arthropod taxa occur in roadside vegetation in urban areas, (2) replacement of exotic woody vegetation by native wildflower meadows can significantly increase arthropod abundance, especially if meadow management permits temporarily unmown areas, and (3) maintenance costs can be considerably reduced by converting woody plantings into wildflower meadows. Considering many groups of arthropods, our study provides new insights into possible measures to support arthropods in urban environments.

## Introduction

A remarkable decline in the number of insect species and in the abundance of insects is currently reported from many places around the world [1–6]. In addition to the considerable loss of organisms that are valuable *per se*, the loss of insects is considered to harm species-interactions [7, 8] and related ecosystem processes [9–11]. As evidence for the decline in insects and public awareness increases, measures are being searched for to stop this development. Besides a more prudent use of agrochemicals, including a reduced application of pesticides and fertilizers, the (re)creation of suitable habitats both in rural and urban areas is being discussed and already realized [12–15].

In the urban environment, the establishment of perennial flower meadows instead of formerly built-up structures or frequently mown lawns is one of the most important measures to promote insects [16–18]. Less obvious, but still relevant, is the improvement of other types of existing urban green spaces [19]. In many cities, the green spaces, including roadside plantings, are dominated by introduced (“exotic”) woody plants, which serve as “distance green” separating different groups of users and may even have some positive effects on human well-being, but less on insects and insect-related processes [20–22], but see [23] for pollinator abundance. An effective measure to improve these green spaces may be to replace the exotic plants by native plants and thereby improve the relationship between green spaces and the regional fauna [24, 25]. Native plants can be trees and shrubs (woody plants), but also forbs and graminoids (herbaceous plants) that are integrated individually or in the form of plant communities into private gardens and public plantings [26].

Flower meadows of native forbs (“wildflowers”) and grasses are increasingly considered as a relevant contribution to the promotion of biodiversity and ecosystem functions in an urban context [27, 28]. In addition to selecting the most suitable plant species, the choice of ecotypes can also play a role in optimizing impacts on biodiversity and ecosystem functions [29, 30]. Although flower meadows can be established on small patches of land, their size and location in relation to harmful (e.g. roads) or beneficial structures (e.g. urban green spaces; natural habitats or larger rural areas as source habitats for species’ colonization) can be important for the establishment and persistence of animal communities using the flower meadows as habitat [31–33].

As predicted by the theory of island biogeography, smaller and more isolated habitat patches (serving as functional habitat islands) are expected to have smaller animal populations and lower species richness [34]. In the urban context, habitat patches such as road islands and roadside plantings separating roads from walkways can be considered as islands that are more or less accessible depending on the mobility of colonizing animals and the distance to source habitats [35, 36]. After colonization, vegetation cover may be decisive in determining whether a species may or may not persist. For animals interacting with plants, not only the presence of a plant is important, but also the size, architecture and persistence of the plant [37, 38].

Mowing, which is necessary for the permanent existence of flower meadows, has a strong impact on the availability of resources (e.g., flowers) and the structural characteristics of meadows [39, 40]. It can have direct and indirect effects on meadow-living animals [18, 41–43]. For example, it is known that many birds and mammals, but also insects, are directly injured by mowing, depending on the mowing techniques used [43, 44]. Indirect effects refer to changes in habitat and resource quality, which include reduced protection from natural enemies and from unfavorable abiotic conditions, and lack of resources [45, 46]. Mowing regimes can therefore be regarded as a fundamental aspect of meadow maintenance, which can be used specifically to increase the conservation value of meadows.

Roadside plantings that are dominated by exotic shrubs need to be cut regularly for safety and aesthetic reasons. The need for regular maintenance work leads to high costs for the responsible authorities, which can seriously affect the economic sustainability of this type of green space vegetation [47, 48]. Given the generally low value of exotic plants for biodiversity and the high maintenance costs, replacing these shrubs with wildflower meadows seems to be a rewarding management measure for urban green spaces. While comparisons of different aspects of biodiversity of frequently mown lawns with flower meadows have already been made in different urban contexts [28, 49, 50], the effects of the conversion of exotic woody roadside vegetation into native flower meadows on the occurrence of insects and other arthropods are not yet known.

Here we have tested these effects of vegetation conversion on arthropod abundance and on maintenance costs in a small city environment in two consecutive years. We compared arthropod numbers in plots covered by the original vegetation, consisting of exotic shrubs (“woody”), with plots covered by intentionally sown wildflower meadows of two different age classes: meadows established five years before the evaluation (referring to study year 1; “old meadow”) and meadows established in the year of the evaluation (referring to study year 1; “young meadow”). In addition to vegetation type (woody, young and old meadows), we considered the size and the location of the plots in terms of distance to the city boundary. We also compared mown and unmown meadow spots that occurred on some of the plots in study year 2.

We addressed the following research questions, considering the conversion of woody roadside plantings into wildflower meadows:

1. Which arthropod taxa are frequently found in urban green spaces?
2. Is there a difference in the abundance of different arthropod groups between flower meadows and woody vegetation, and which arthropod groups benefit from flower meadows and which from woody vegetation?
3. Is the abundance of arthropod groups influenced by the size or location of green spaces, the age of the flower meadow or the mowing regime?
4. Can the conversion of woody vegetation into flower meadows help to reduce maintenance costs?

## Materials and methods

### Study sites

**Fig 1:**
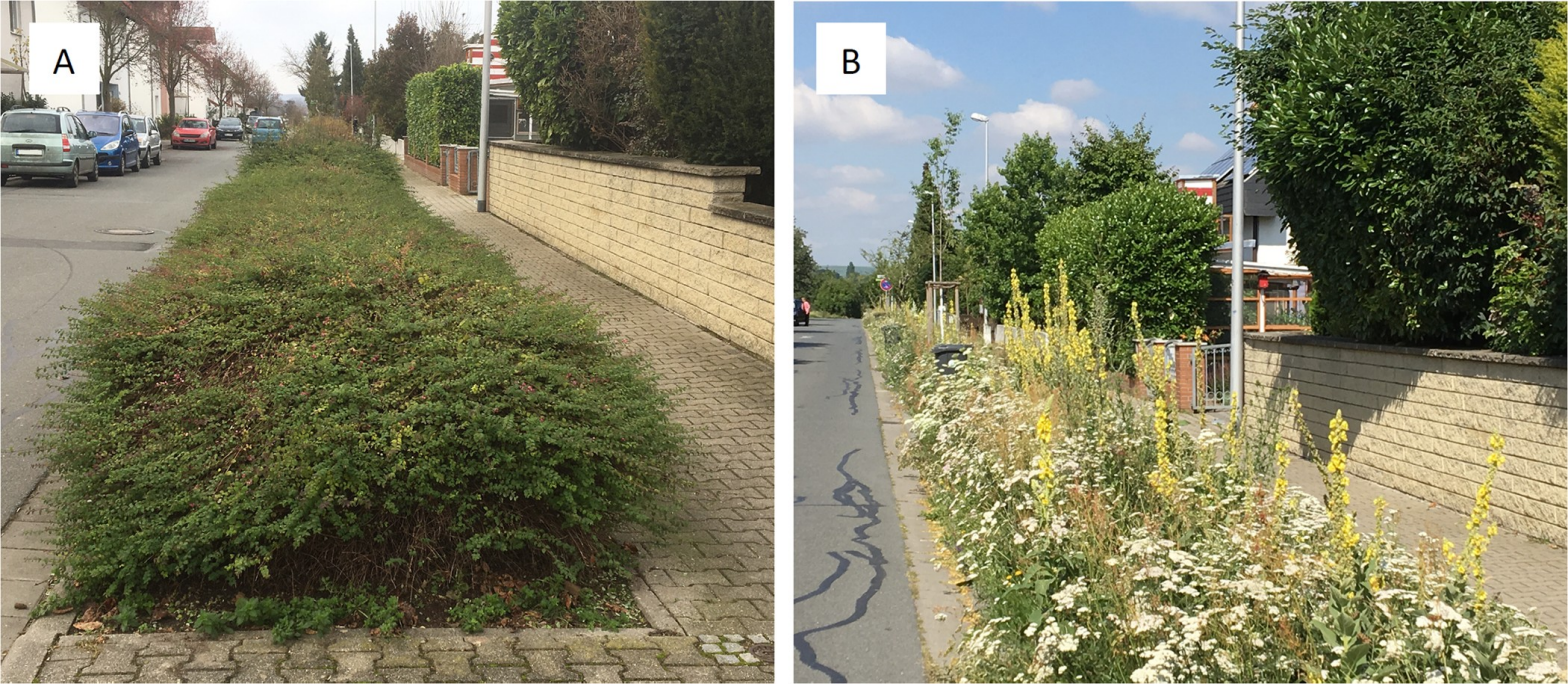
Example of original woody roadside vegetation (A), and a newly established wildflower meadow (B).

The study was conducted in the administrative area of Riedstadt (24.202 inhabitants, 74 km² municipal area) in southwest Germany (49°50′14″N, 08°30′16″E). Riedstadt consists of five formerly independent municipalities and lies on the border of the Rhine-Main metropolitan region. In 2009/2010, after approval by the city council, the administration of Riedstadt began to convert areas of roadside vegetation consisting of exotic woody plants (including *Symphoricarpus chenaultii* “Hancock”, *Mahonia aquifolium*, *Lonicera nitida* and *L*. *pileata*, as well as various forms of *Cotoneaster* spp.) into wildflower meadows (Fig 1). The woody vegetation was removed and the often compacted and weedy (e.g. underground runners of *Mahonia aquifolium* and *Elymus repens*) soil was replaced by a nutrient-poor mineral substrate with almost no organic materials (organic components < 1%). After thus preparing the ground, a mixture of up to 41 native forb species of certified regional origin and some additional geophytes–all plants were selected from a total pool of 70 species (S2 Table)–was sown or planted per plot. In the years 2011 to 2019, the conversion work was continued. To support the development of species-rich wildflower meadows, the new meadows are mown twice a year, in June/July and at the end of February, shortly before the start of the new vegetation period. Whereas the first cut is removed from the plots, the second cut is mulched and remains on the plot to achieve a compromise between the goal of “increasing biodiversity” (by removing the first cut with mostly high vegetation biomass to prevent nutrient accumulation) and the goal of “reducing the costs” (mulching the second, mostly rather sparse growth). About 5–10% of the meadow area usually remains unmown to provide refuges for invertebrates [51, 52].

### Arthropod sampling

We sampled arthropods in the newly created wildflower meadows and in the original woody roadside plantings in 40 plots in 2015 (year 1) and in 41 plots in 2016 (year 2). Two plots with original vegetation studied in year 1 were modified by construction work and were replaced by two other plots in year 2. The studied wildflower plots had been converted in February 2010 (“old meadow”, 20 plots; 21 plots in year 2), or in March 2015 (“young meadow”, 10 plots). Plots with original woody vegetation served as control (“woody”, 10 plots). The plots were located in different districts of Riedstadt municipalities and differed in size (between 3.3 and 1362 m^2^) and distance to the city boundary (between 1 and 273 m linear distance to rural area such as farmland, meadows and forest). In year 2, nine of the studied meadow plots were mown at the end of June, with the exception of 5–10% of the area that remained unmown. We used these plots to assess the potential influence of the mowing regime (“mown meadow” vs. “unmown meadow”) on arthropod density.

We compared arthropod numbers between woody and wildflower plots using two different sampling methods. In year 1, we quantified arthropod “activity abundance” [53] with pitfall traps [54, 55]. We set up two pitfall traps per plot, one in the plot center and one near the edge of the plot, at a distance of 50 cm from the road. As trap containers we used circular plastic cups (diameter 9.5 cm; height: 10 cm; volume: 500 ml) (see [56] for efficiency of circular traps) and inserted dome lids with a hole (diameter 3 cm) as funnels to reduce the contamination by loose plant material and to minimize small vertebrate by-catch [57]. Pitfall traps were filled with 150 ml of water with odorless detergent as trapping liquid and operated for 24 hours on five sampling events between 9 June and 16 July. After 24 hours, all arthropods were removed from the traps and stored in 70% ethanol for further processing. In year 2, we quantified arthropod density by suction sampling within a “biocenometer”, an aluminium frame covered with gauze (1 m × 1 m area, height 0.6 m). The biocenometer is quickly placed on an area to prevent arthropods from escaping prior to sampling [58]. The biocenometer ensured the sampling of all arthropods from 1m^2^ areas in the center of our study plots. In woody vegetation, the biocenometer was gently pushed into the vegetation, and the vegetation inside the cage, the vegetation down to the ground, and the soil surface were vacuumed. Biocenometer sampling was conducted once on all plots on a sunny summer day (25 July). To assess the possible effects of the mowing regime on arthropod occurrence, we took one biocenometer sample from a mown (mowing took place four weeks prior to biocenometer sampling) and one from an unmown spot of those plots that had mown and unmown meadow fractions. Mobile flower visitors, including bees and butterflies, constantly switch between flowers and show a strong dependence on current weather conditions, so that they are not recorded representatively by the applied biocenometer technique. As many bee and butterfly species are also protected by law, they were not vacuumed or released immediately after sampling and were not included in our analyses. The remaining arthropods were anesthetized with CO_2_ and frozen until further processing. Samples from year 1 and year 2 were sorted to higher taxa levels (S1 Table) and all individuals belonging to these taxa were counted. Permission to enter the study areas and to collect data was granted by the City of Riedstadt, Department of the Environment.

### Maintenance and conversion costs

To interpret the cost information correctly, it should be pointed out that the maintenance costs presented here refer only to the specific conditions in Riedstadt. The location, shape, size and type of vegetation of the area, the availability of manpower (gardeners and/or workers) and equipment as well as the costs for transport and material disposal affect the maintenance costs. The costs/m^2^ given here in euros are based on the working time required to maintain specified vegetation types per year. They thus allow a comparison of costs before and after the conversion of inner-city green spaces, but are not directly comparable with the maintenance costs incurred in other cities or provided by professional horticultural enterprises. In addition to the maintenance costs, the costs for the conversion of the original woody vegetation into wildflower meadows are also shown. These costs include material costs, external services and services provided by the city's workers.

All urban green spaces in Riedstadt are maintained by urban gardeners and workers. The maintenance costs were calculated on the basis of the working time for the maintenance of the plots of the different types of vegetation for the whole period for which data are available: 2010 to 2018 for the oldest meadows, less for younger meadows; average costs over a period of five years before the conversion for woody vegetation.

### Statistical analysis

Pitfall trap samples from the same plot were pooled across the two trap positions (arthropod numbers did not differ significantly between the plot center and edge; S3 Table) and the five sampling events to compensate for short-term weather-related fluctuations in arthropod activity during the study period and to reduce the influence of outliers [55]. As some pitfall traps were destroyed at individual sampling dates (12 of 400 traps were lost), the pooled arthropod numbers per plot were standardized to the number of usable traps (ranging between seven and ten per plot for all five sampling events). We used linear mixed effects models (LME; using the *lme()* function of the *nlme* package [59]) to analyze the effects of vegetation type, plot size and plot distance to the city boundary on the standardized activity abundance (pitfall traps) or density (biocenometer) of arthropod groups represented by at least 98 individuals (i.e., the total number of Orthoptera) summed across all samples in both study years. The 13 arthropod taxa thus selected represented 97% of all arthropods sampled in both study years (S1 Table). Arthropod abundances were square root transformed where necessary to account for heteroscedasticity. All LMEs for individual taxa contained “plot ID” nested in “district” as a random effect to account for the nested design. Individual effects of vegetation type were then analyzed using one-way ANOVA and Tukey-HSD post-hoc tests on the LMEs. We used the *glht()* function of the *multcomp* package [60] for the post-hoc tests. The strength and direction of effects from continuous variables (plot size and distance to city boundary) were obtained from estimates in model summaries. Spearman rank correlation was used to evaluate the relationship between abundance and incidence of taxa in the same plots, and between incidences of taxa in the two study years. To assess the effects of mowing regime, we used paired t-tests or paired samples Wilcoxon tests (when assumptions of normality and heteroscedasticity of t-tests were not met following square root transformation of data) for the subset of meadow plots that contained both mown and unmown spots. All statistical analyses were performed with R version 3.6.2 [61].

## Results

### Overview on arthropod abundance

During our study we collected more than 27,000 individuals of arthropods in plots of urban roadside vegetation. The collected arthropods represented the major arthropod taxa occurring in Germany, but different taxa dominated the samples depending on the sampling method (Tables 1 and 2). Opiliones, Collembola, Aphidoidea, and Formicidae were proportionally more abundant in pitfall traps (year 1) than in suction samples (year 2), whereas Araneae, Orthoptera, Auchenorrhyncha, Heteroptera, Coleoptera and Brachycera were proportionally more abundant in suction samples than in pitfall traps. The total number of arthropods collected ranged between 2 and 710 individuals for single pitfall traps (Table 1), and between 12 and 721 individuals/m^2^ for single suction samples (Table 2). The incidence in individual samples or in plots as a measure of commonness in the different plots varied strongly between individual taxa (Tables 1 and 2). Generally, more abundant taxa were also found in more plots, although the correlation between abundance and incidence was significant only in year 1 (year 1: r_s_ = 0.945, *P* < 0.0001; year 2: r_s_ = 0.524, *P* = 0.066; N = 13). Araneae (68%), Collembola (86%) and especially Formicidae (91%) occurred in most pitfall traps. In suction samples, Formicidae (92%), Heteroptera (94%), Araneae (98%), Auchenorrhyncha (98%), Coleoptera (98%), Brachycera (100%) and Apocrita (100%) were most regularly found. Considering the occurrence in plots, in year 1 most taxa (9 out of 13) were sampled in at least 90% of plots and five taxa in all plots (Araneae, Collembola, Aphidoidea, Coleoptera, Formicidae). Only Orthoptera were found in less than half of the plots, with the suborders Caelifera accounting for 87% and Ensifera for 13% of all collected Orthoptera individuals. In year 2, seven taxa occurred in more than 90% of the plots, two taxa in all plots (Brachycera, Apocrita) and three taxa in less than 50% (Opiliones, Isopoda and Nematocera). The Orthoptera were more evenly represented by Caelifera (51% of individuals) and Ensifera (49% of individuals) than in year 1. The incidence of taxa in plots was not significantly correlated between year 1 and year 2 (r_s_ = 0.310, *P* = 0.302; N = 13).

**Table 1:** Overview on arthropods sampled by pitfall traps in study year 1.

	OPIL	ARAN	ISOP	COLL	ORTH	APHI	AUCH	HETE	COLE	NEMA	BRAC	APOC	FORM	TOTAL
Total number	113	512	288	7193	31	2022	659	360	398	98	196	110	4864	17396
Incidence in samples (%)	19	66	20	86	7	55	52	45	44	16	33	21	91	100
Incidence in plots (%)	63	100	78	100	38	100	93	98	100	85	93	90	100	100
Maximum number per sample	6	12	30	697	3	268	18	13	32	11	13	4	107	710
Mean number (±SE) per sample woody	0.55 (0.11)	0.87 (0.10)	0.28 (0.09)	3.91 (0.48)	0.01 (0.01)	0.86 (0.43)	0.55 (0.12)	0.29 (0.06)	1.33 (0.40)	0.46 (0.14)	0.39 (0.07)	0.38 (0.07)	6.82 (1.00)	17.36 (1.71)
Mean number (±SE) per sample meadow	0.20 (0.03)	1.47 (0.09)	0.90 (0.19)	23.48 (3.11)	0.10 (0.02)	6.68 (1.26)	2.09 (0.17)	1.14 (0.10)	0.92 (0.09)	0.18 (0.03)	0.54 (0.07)	0.25 (0.04)	14.47 (1.06)	54.12 (3.70)
Change (%) woody to meadow	-63	70	227	501	914	680	279	301	-30	-60	41	-33	112	212

OPIL: Opiliones, ARAN: Araneae, ISOP: Isopoda, COLL: Collembola, ORTH: Orthoptera, APHI: Aphidoidea, AUCH: Auchenorrhyncha, HETE: Heteroptera, COLE: Coleoptera, NEMA: Nematocera, BRAC: Brachycera, APOC: Apocrita, FORM: Formicidae, TOTAL: total of all arthropods sampled by pitfall traps in year 1.

**Table 2:** Overview on arthropods sampled by suction sampling in study year 2.

	OPIL	ARAN	ISOP	COLL	ORTH	APHI	AUCH	HETE	COLE	NEMA	BRAC	APOC	FORM	TOTAL
Total number	31	1017	124	1259	67	253	999	1431	1196	78	1067	545	1483	9843
Incidence in samples (%)	34	98	34	60	60	54	98	94	98	30	100	100	92	100
Incidence in plots (%)	41	98	34	59	63	59	98	93	98	34	100	100	90	100
Maximum number per sample (1m^2^)	5	60	22	487	8	60	70	171	97	21	197	42	251	721
Mean number (±SE) per sample woody	0.40 (0.16)	11.10 (2.72)	1.10 (1.10)	3.20 (1.47)	0.90 (0.28)	0 (0)	4.70 (1.46)	2.70 (0.84)	5.20 (1.53)	3.30 (1.05)	6.00 (1.01)	5.30 (1.21)	5.10 (2.18)	57.10 (9.84)
Mean number (±SE) per sample meadow	0.63 (0.23)	19.87 (2.42)	2.50 (0.94)	33.00 (17.76)	1.53 (0.39)	7.40 (2.26)	18.80 (2.02)	23.93 (2.96)	23.83 (2.73)	0.43 (0.21)	17.57 (2.43)	12.33 (1.84)	38.40 (9.94)	205.37 (24.58)
Change (%) woody to meadow	58	79	127	931	70	NA	300	786	358	-87	193	133	653	260
Mean number (±SE) mown meadow	0.33 (0.33)	19.22 (3.07)	3.00 (1.86)	23.56 (17.05)	0.89 (0.35)	5.00 (1.76)	21.33 (3.83)	25.22 (5.06)	17.22 (2.62)	0.44 (0.34)	17.33 (5.48)	11.89 (2.08)	30.89 (12.64)	180.00 (22.99)
Mean number (±SE) unmown meadow	0.78 (0.43)	29.22 (5.42)	4.22 (2.34)	24.44 (9.93)	1.22 (0.40)	3.11 (1.39)	41.44 (6.21)	65.78 (19.38)	45.33 (8.66)	2.44 (2.32)	31.44 (9.99)	11.67 (2.37)	27.44 (9.96)	294.22 (34.55)
Change (%) mown to unmown meadow	133	52	41	4	38	-38	94	161	163	450	81	-2	-11	63

OPIL: Opiliones, ARAN: Araneae, ISOP: Isopoda, COLL: Collembola, ORTH: Orthoptera, APHI: Aphidoidea, AUCH: Auchenorrhyncha, HETE: Heteroptera, COLE: Coleoptera, NEMA: Nematocera, BRAC: Brachycera, APOC: Apocrita, FORM: Formicidae, TOTAL: total of all arthropods sampled by suction sampling in year 2. “Mean number per sample meadow” refers to mown meadow spots only, other values to mown and unmown meadow spots; comparison of mown and unmown meadows refers only to plots containing a mown and an unmown meadow spot.

### Influence of vegetation type on arthropod abundance

**Fig 2:**
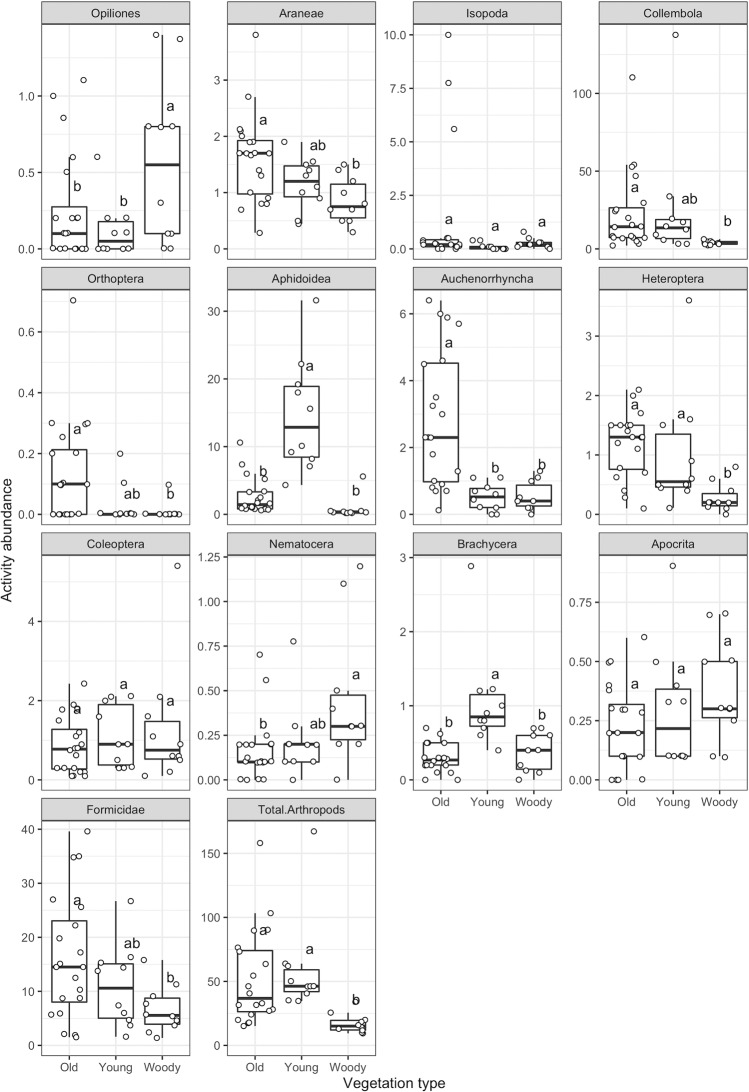
Activity abundance of arthropod taxa in different urban vegetation types in study year 1. Each data point represents the number of individuals for the respective arthropod taxon per plot sampled in pitfall traps, standardized by the number of operative traps. Old: meadows established five years before arthropod sampling; Young: meadows established in the year of arthropod sampling; Woody: original woody roadside vegetation consisting of different exotic shrubs; different letters above boxes indicate significant differences (ANOVA followed by Tukey post-hoc test; *P* < 0.05).

**Fig 3:**
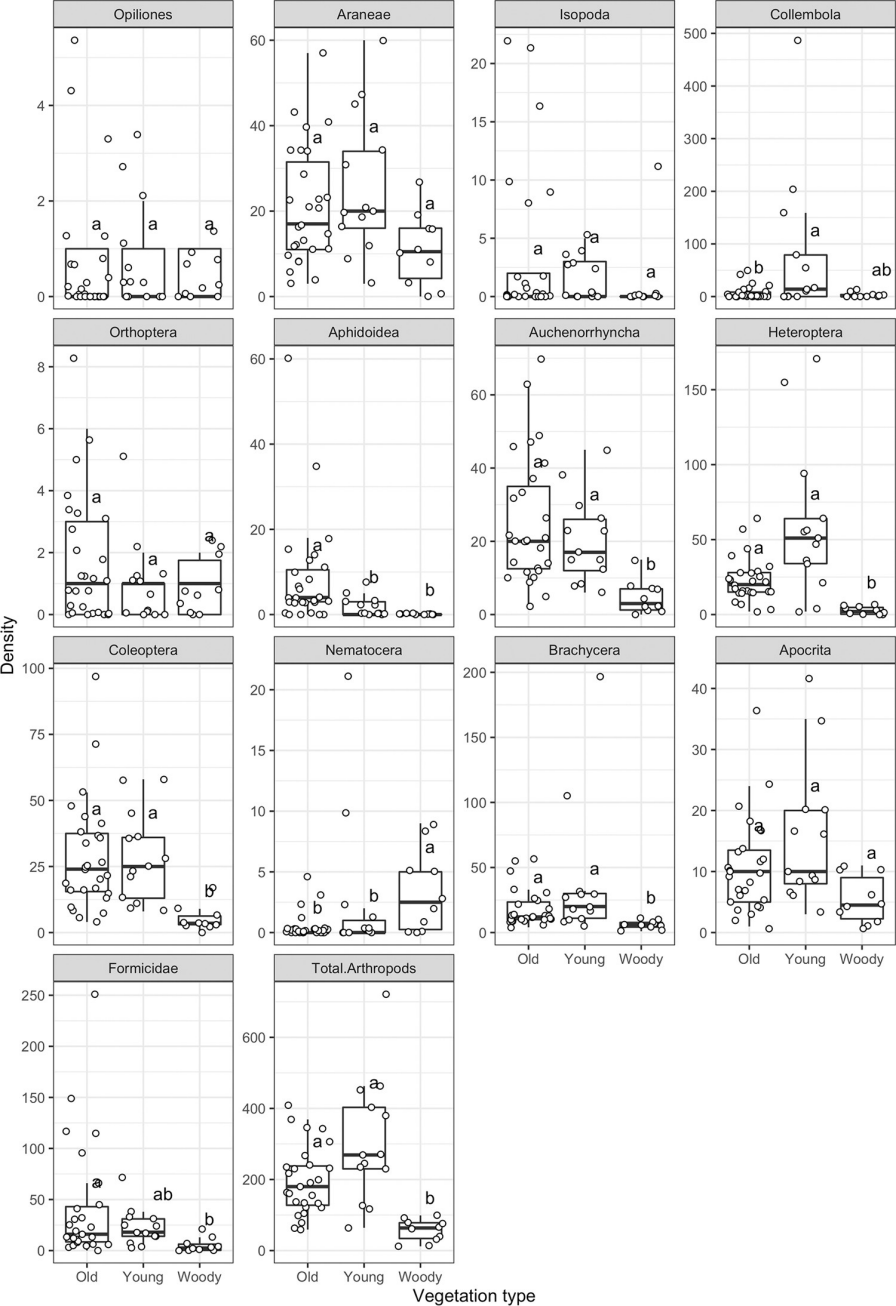
Density of arthropod taxa in different urban vegetation types in study year 2. Each data point represents the number of individuals for the respective arthropod taxon per plot assessed by suction sampling from a defined area within a “biocenometer” (gauze-covered aluminium frame; 1 m x 1 m area, height 0.6 m). Old: meadows established six years before arthropod sampling; Young: meadows established one year before arthropod sampling; Woody: original woody roadside vegetation consisting of different exotic shrubs; different letters above boxes indicate significant differences (ANOVA followed by Tukey post-hoc test; *P* < 0.05).

Comparing the arthropod numbers in meadows and woody roadside vegetation, we found for most, but not all, arthropod taxa a markedly higher number in meadows. The total number of collected arthropods in meadows was 212% higher than in woody vegetation in year 1 (Table 1), and 260% higher in year 2 (Table 2). The average arthropod number in pitfall traps was 54.1 (±3.7 SE) in meadows and 17.4 (±1.7) in woody vegetation (Table 1). In suction samples, arthropod density/m^2^ was 231.8 (±21.5) for meadows and 57.1 (±9.8) for woody vegetation (Table 2). Despite strong variation in arthropod numbers between plots belonging to the same vegetation type, the vegetation type showed a significant influence on 10 out of 13 arthropod groups in pitfall traps (only Isopoda, Coleoptera and Apocrita did not differ significantly; Fig 2 and Table 3). In suction samples, 9 out of 13 arthropod groups were significantly influenced by vegetation type (Fig 3 and Table 3). For those taxa that were significantly affected by the vegetation type (*P* < 0.05, Table 3), the increase in numbers between woody vegetation and meadows ranged between 41% and 914% for pitfall traps (Table 1), and between 133% and 931% for suction samples (Table 2). A significant decline of individual numbers from meadows to woody vegetation was observed only for Nematocera (mainly mosquitoes; 60% decline in year 1, 87% decline in year 2).

**Table 3:** Influence of size of green space plots, distance to city boundary and vegetation type on activity abundance (year 1) or density (year 2) of different arthropod taxa in urban green spaces. Influence was assessed by linear mixed effects models (LME) for standardized abundance of arthropod groups that were represented by at least 98 individuals (Orthoptera) summed up across all samples in both study years.

		Year 1			Year 2		
		SIZE	DISTANCE	TYPE	SIZE	DISTANCE	TYPE
	numDF	1	1	2	1	1	2
	denDF	31	31	31	31	31	31
**Opiliones**	***F***	0.935	0.713	3.883	0.083	1.231	0.009
	***P***	0.341	0.405	**0.031**	0.776	0.276	0.991
**Araneae**	***F***	0.269	1.036	4.089	0.008	0.107	3.056
	***P***	0.608	0.317	**0.027**	0.928	0.746	0.061
**Isopoda**	***F***	0.090	0.049	1.972	0.420	0.384	0.615
	***P***	0.767	0.827	0.156	0.522	0.540	0.547
**Collembola**	***F***	1.089	0.632	3.913	0.510	1.988	3.937
	***P***	0.305	0.433	**0.031**	0.480	0.168	**0.030**
**Orthoptera**	***F***	0.314	0.003	5.366	0.430	0.009	0.064
	***P***	0.579	0.954	**0.010**	0.517	0.923	0.938
**Aphidoidea**	***F***	9.374	3.908	31.098	0.557	1.111	11.412
	***P***	**0.005**	0.057	**<0.001**	0.461	0.300	**<0.001**
**Auchenorrhyncha**	***F***	0.386	0.883	19.280	2.751	0.324	11.266
	***P***	0.539	0.355	**<0.001**	0.107	0.573	**<0.001**
**Heteroptera**	***F***	2.055	2.540	7.492	1.582	0.209	18.109
	***P***	0.162	0.121	**0.002**	0.218	0.651	**<0.001**
**Coleoptera**	***F***	0.001	12.355	1.237	0.018	0.135	14.193
	***P***	0.976	**0.001**	0.304	0.894	0.716	**<0.001**
**Nematocera**	***F***	1.122	11.693	3.375	3.753	2.943	5.913
	***P***	0.298	**0.002**	**0.047**	0.062	0.096	**0.007**
**Brachycera**	***F***	0.610	1.591	13.826	1.337	3.175	7.489
	***P***	0.441	0.217	**<0.001**	0.256	0.085	**0.002**
**Apocrita**	***F***	3.420	0.041	2.301	0.001	0.673	3.446
	***P***	0.074	0.840	0.117	0.979	0.418	**0.045**
**Formicidae**	***F***	0.023	0.318	4.723	0.840	0.014	4.597
	***P***	0.880	0.577	**0.016**	0.366	0.908	**0.018**
**Total arthropods**	***F***	1.380	0.058	8.868	0.993	0.157	11.853
	***P***	0.249	0.811	**0.001**	0.327	0.695	**<0.001**

F-values and *P*-values taken from ANOVA, significant *P*-values (at 0.05) are in bold.

### Influence of meadow age, plot location and plot size on arthropod abundance

Besides differences between meadows and woody vegetation, we found that the arthropod numbers, especially in year 1, were partly influenced by meadow age. In year 1, Orthoptera and most notably Auchenorrhyncha were more numerous in old meadows, whereas Aphidoidea and Brachycera were especially abundant in young meadows (Fig 2). In year 2, the differences in arthropod communities between young and old meadows were less pronounced, and no significant differences in density between meadow types were detected for any taxon (Fig 3).

We found no strong influence of green space size or distance to the city boundary on numbers of most arthropod taxa (Table 3). The only significant effect of green space size was detected for Aphidoidea in year 1 (Table 3), with no clear direction of this effect (model estimate = 0.000, SE = 0.001). The distance to the city boundary affected the abundance of Coleoptera and Nematocera in year 1. For both taxa, the numbers increased from the boundary towards the city center (Coleoptera: estimate = 0.004, SE = 0.001; Nematocera: estimate = 0.001, SE = 0.001).

### Influence of mowing on arthropod abundance

**Fig 4:**
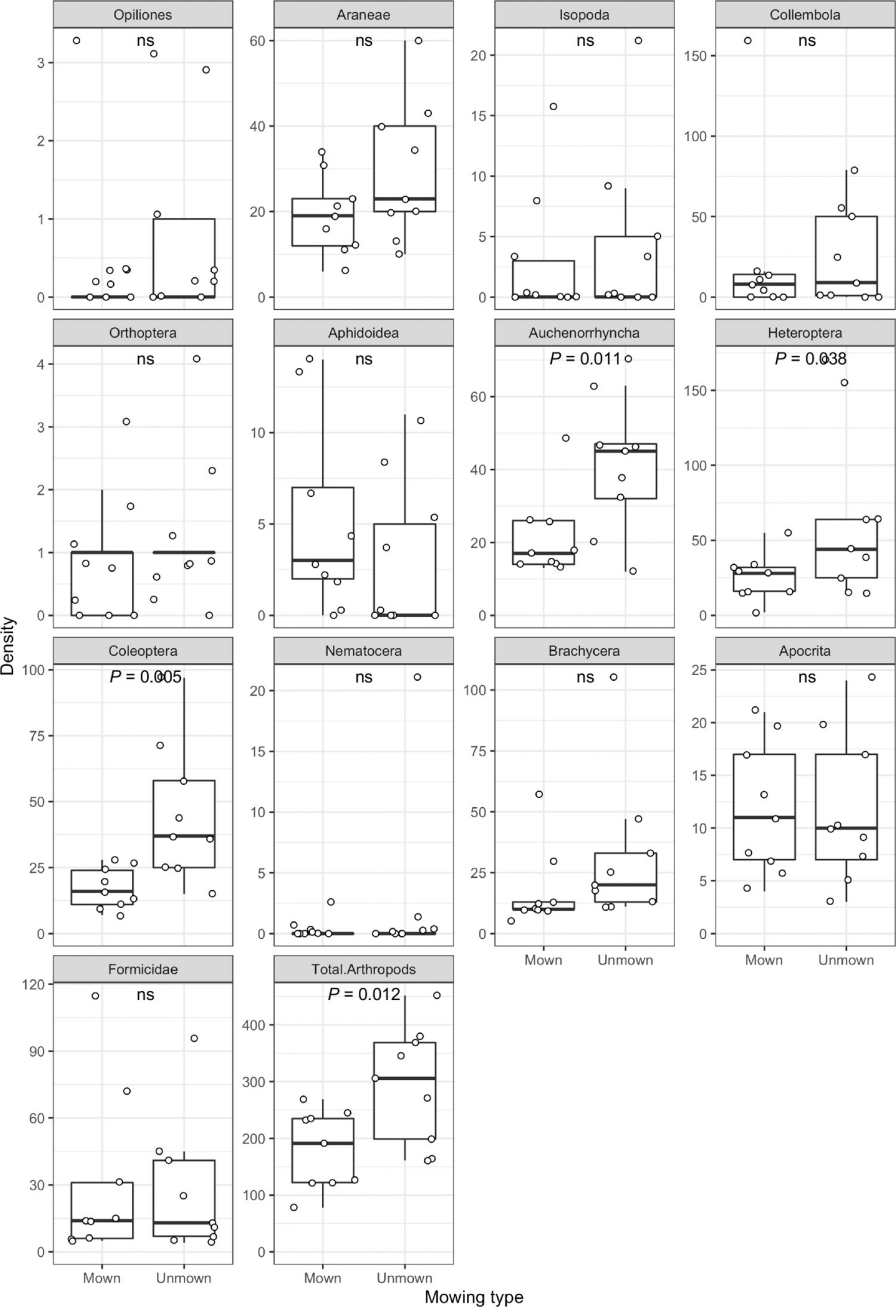
Density of arthropod taxa in mown and unmown urban meadow spots. Arthropods were assessed by suction sampling from a defined area within a “biocenometer” (gauze-covered aluminium frame; 1 m x 1 m area, height 0.6 m). Each data point represents the number of individuals for the respective arthropod taxon per spot, with one mown and one unmown spot per plot. Statistical comparisons were conducted by paired t-tests or Wilcoxon tests; ns: not significant.

We found that unmown meadow spots generally contained markedly more arthropod individuals than mown spots (increase in total arthropod numbers 63%; t = 3.21, *P* = 0.012; N = 9; Fig 4 and Tables 2 and S4). However, mowing did not affect all arthropod taxa equally (Fig 4 and Table 2). Unmown meadow spots contained significantly more individuals of Auchenorrhyncha (W = 44, *P* = 0.011), Heteroptera (t = 2.48, *P* = 0.038) and Coleoptera (t = 3.88, *P* = 0.005), whereas other taxa were not significantly affected (Fig 4 and S4 Table).

### Costs for maintenance and vegetation conversion

The average maintenance costs for plots with woody vegetation in Riedstadt were more than five times higher than the maintenance costs for meadows (Table 4). The costs for the conversion of woody into meadow areas amounted to 38.4 euros/m^2^, divided into 14.2 euros for material costs and external services and 24.2 euros for the services provided by the city's workers. Taking into account the annual cost savings of almost 5.5 euros/m^2^ for the maintenance of wildflower meadows compared to woody vegetation (Table 4), the conversion costs paid for themselves within seven years. The values given are average values across green spaces differing in size. Internal estimates (Matthias Harnisch) of the role of green space size for maintenance costs indicate that maintaining small meadow areas is more expensive than maintaining larger areas (example estimate: 1 x 100m^2^ area: 1.03 €/m^2^; 20 x 5m^2^: 1.55 €/m^2^), but costs also depend on accessibility and distance between plots. Without the relatively high conversion costs, the cost of establishing flower meadows is much lower. For Riedstadt, the approximate cost estimates range between 0.42 and 0.82 €/m^2^ for the conversion of intensively mown lawns (8–12 mowing operations per year) into wildflower meadows using certified seeds of regional provenance (costs include rotary tilling of lawns and seeds of wild meadow plants; price differences relate to different seed mixtures).

**Table 4:** Maintenance costs of different types of urban green space vegetation. Maintenance includes regular cutting of woody vegetation, mowing of meadows in summer and late winter, and removal of plant material. Costs are the costs for maintenance of woody green space before conversion (five years average) and average (± SE) annual costs for maintenance of flower meadows in five municipalities belonging to the city of Riedstadt. The years in brackets indicate the years for which information on the maintenance of the meadows has been available since the meadows were established.

Municipality	Costs (EUR) per m^2^ woody vegetation	Costs (EUR) per m^2^ flower meadow
Erfelden (2010–2018)	5.52	1.54 (0.17)
Goddelau (2013–2018)	8.93	0.90 (0.20)
Wolfskehlen (2013–2018)	7.94	0.86 (0.14)
Leeheim (2015–2018)	6.00	2.31 (0.55)
Crumstadt (2017–2018)	5.49	0.85 (0.12)
Average	6.78 (0.70)	1.29 (0.29)

## Discussion

Our study showed that the roadside vegetation in urban areas can serve as a habitat for a large variety of arthropod taxa and that the replacement of exotic woody vegetation by native herbaceous vegetation can markedly increase the numbers of arthropods living in urban green spaces irrespective of the size and isolation of these areas. Besides these effects of vegetation conversion, our study also showed that meadow age and mowing status can strongly influence the occurrence of different arthropod taxa. With regard to economic sustainability, our study emphasizes that the conversion of formerly intensively managed urban vegetation to wildflower meadows can contribute to effectively reducing the costs of green space maintenance.

The conversion of urban roadside vegetation from exotic woody vegetation to native flower meadows influenced the numbers of arthropod individuals of a variety of arthropod taxa, with most taxa profiting from flower meadows in terms of increased activity abundance or density. In the urban context, positive effects of flower meadows compared to mown lawns have been reported so far for flower visitors and pollinators [17, 62]. Higher arthropod numbers were also reported for urban meadows compared to short-mown grassland [16]. A comparison with exotic woody plant vegetation has not yet been carried out to our knowledge. Whereas the value of urban woody plantings for birds is well known [63–65], the value of these plantings for arthropod biodiversity does not seem to be equally known, although some studies suggest a higher value of native than exotic shrubs for plant-living invertebrates [24, 66, 67]. In this sense our findings of higher numbers of arthropods on wildflower meadows than on woody plantings should also be compared with the occurrence of arthropods on urban woody plantings consisting of native species [68].

Our study showed the strongest effects on arthropod numbers being related to vegetation conversion, but we also found effects of meadow age and mowing regime, and in a few cases effects of green space distance to the city boundary and of the size of the green spaces. These effects are now only taken up briefly to create a general context. In the following, the individual taxa are then discussed in more detail.

A positive influence of the age of green spaces on the species richness of arthropods was described for other cities [36, 69] and was explained by the creation of more ecological niches due to progressing succession and increasing probability of a successful stochastic local immigration. In our study, the effects of meadow age were expressed as arthropod numbers differing between old and young meadows. In year 1, differences could be expected as newly created meadows were only sparsely vegetated and showed only a limited number of established plant species and individuals, and arthropods had little time to colonize the new meadow plots [70, 71]. Accordingly, in year 1 we found higher individual numbers of most taxa on old compared to young meadows and only in two taxa (Aphidoidea, Brachycera) significantly higher numbers on young meadows. In year 2, the young meadow plots were completely vegetated and many plants bloomed, which provided food resources for flower- and fruit-feeding insects and their predators. As a result, differences in arthropod numbers between young and old meadows were generally less pronounced in year 2 than in year 1 for most taxa.

The distance to the city boundary can have profound effects on taxa that colonize urban environments from rural or forested land outside the urban area. In such cases, proximity to source habitats is expected to be associated with higher species and also individual numbers, whereas numbers decrease towards the city center [35, 36, 72, 73]. However, in cases where rural areas outside cities are characterized by intensive agriculture or industrialization, negative influences such as the influx of contaminants like pesticides can counteract positive effects of proximity to potential source populations [74, 75]. In intensively farmed rural areas, the populations of most arthropod species may also be greatly reduced, which limits the source effect of such areas [3, 5]. Our finding of a generally weak influence of the distance to the city boundary may be explained by the relatively short distances considered in our study. The impact may well be higher as the size of the city increases.

The habitat size is generally positively linked to the number of species based on positive species-area relationships [76]. Urban habitats are usually islands in a matrix of more or less hostile environments for most animal and plant species [77]. Besides the area size, the heterogeneity of environmental conditions in island patches and the connectivity to other patches is important [78], which can reduce the pure area size effects and mitigate negative influences related to fragmentation and isolation [79, 80]. In contrast to species number, the positive effect of area size on the density of individuals strongly depends on the specific taxa under consideration [81].

### Specific determinants of arthropod abundance

Depending on their habitat requirements and life history, the replacement of woody vegetation by flower meadows may have differing effects on members of different arthropod taxa. This may even be true for those taxa that responded in comparable ways to the vegetation conversion. As in addition different arthropod taxa are differently well represented by the two sampling methods applied in our study, we will address each arthropod taxon separately to discuss our findings with regard to effects of vegetation conversion, meadow age and mowing regime.

#### Opiliones

Opiliones (harvestmen) [82] were relatively rare in all vegetation types studied, which is consistent with other studies showing that Opiliones are not very common in urban green spaces [16]. Opiliones were found more frequently in repeated pitfall samples than in one-time suction samples. Pitfall traps revealed a higher number and more regular occurrence in woody compared to meadow vegetation, especially in newly established meadows. This is in line with other studies from urban environments, which reported higher numbers of Opiliones in urban forest fragments than in vacant lots or community gardens [83]. The suction samples did not confirm this finding, but showed comparable densities per surface area for the different vegetation types. Higher catches in pitfall traps in woody vegetation could therefore either reflect a higher activity abundance in this habitat type or simply a higher accessibility of pitfall traps in woody vegetation compared to dense vegetation on the ground surface of meadows. Accessibility alone is probably not the only reason for higher catches in pitfall traps in woody vegetation as the lowest catches in traps were obtained in young meadows, which were characterized by many open areas and the lowest vegetation density. Low numbers in the meadows were not strongly affected by the mowing regime, as we found no clear differences between mown and unmown meadow spots. In other urban areas it was found that activity abundance of Opiliones was greater in habitats with shorter vegetation [84], and that they occurred more commonly in vacant lots (vegetated with grasses and flowering forbs, monthly mown) than in newly created urban gardens [84]. In general, Opiliones need cover (as found in stacks of birch logs: [85]) and avoid harsh climatic conditions, which may explain their low number especially in young meadows in year 1. As “active hunters that forage on the soil surface as well as within plant canopies”[84], they may not strongly benefit from flower meadows in terms of considerably improved prey availability. Comparing native with exotic vegetation, Opiliones tended to be more abundant (though not significantly) in native birch *Betula pendula* compared to non-native black locust *Robinia pseudacacia* pioneer woodlands on urban sites in Berlin, Germany [86].

#### Araneae

Araneae (spiders) were abundant and frequently found by both sampling methods in all vegetation types. Pitfall trapping revealed much higher numbers on old flower meadows than in woody vegetation, suggesting that cursorial (wandering) species, which are well represented by pitfall trapping [87], benefited from flower meadows. This finding supports other studies, which found that typical groups of cursorial spiders such as lycosids and gnaphosids occur at high activity abundances in grassy areas [88]. Suction sampling, which can be assumed to equally assess cursorial and web-building spiders, did not show strong differences between vegetation types. It is possible that the higher number of cursorial species in meadows is partially compensated by an increased number of web-building spiders in the spatially more complex environment of woody vegetation. A higher number of spiders due to increased vegetation complexity is also indicated by the (non-significant) increase of spider numbers in unmown compared to mown meadows. In the case of mowing, stronger effects were to be expected, as other studies showed clear differences between meadows differing in mowing intensity [89].

#### Isopoda

Isopoda (woodlice) were generally rare in most plots, although they occurred–according to pitfall trap sampling–in the majority of old meadow and woody plots, but less so in young meadow plots. Rarity in young meadows may be related to the low vegetation cover [90], but also to the low dispersal capacity of Isopoda in an urban context [91]. Suction sampling revealed fewer individuals and a lower incidence in plots than pitfall traps, which can be related to difficulties in sampling these predominantly nocturnal organisms in short-term day samples. In urban green spaces [16], Isopoda generally may occur less frequently as in other, more suitable habitats, including deciduous forests or calcareous grassland and heath, from which densities/m^2^ of 500 to 1500 individuals were reported [92], with a described maximum density of 7900 individuals [93]. However, Isopoda can also be a dominant group of soil macrofauna in city parks and gardens [94, 95]. Although we have found no effect of mowing in terms of differing numbers of Isopoda in mown versus unmown meadow spots, we suggest that pitfall studies should be conducted to better assess this question. In mown meadows, further processing of plant material may influence the occurrence of soil arthropods, with a negative mulching effect for Isopoda numbers [96]. As in other studies [81], we found no influence of the size of green spaces on the number of Isopoda.

#### Collembola

Collembola (springtails) were the most abundant arthropod taxon in pitfall traps and the third most abundant taxon in suction samples. They occurred in all vegetation types and plots (based on pitfall traps), but were consistently more common in meadows than in woody vegetation. Apart from the vegetation type, no other factors investigated had a significant influence on Collembola numbers. High abundances of Collembola were also reported from other studies on urban green space invertebrates [16, 97]. The finding of a higher number in meadows compared to woody vegetation was not to be expected, since it is known that Collembola can reach a higher density in leaf litter and forest soils than in meadows [98, 99] and even benefit from the presence of single trees [100]. One possible explanation for the lower Collembola numbers is that the woody roadside plantings of very dense, exotic shrubs did not produce a valuable litter and climatically suitable habitat for forest species, nor were they particularly suitable for species of open habitats [101]. The compacted and dry soil beneath the dense shrubs may also be only a suboptimal habitat for predominantly soil-living organisms such as Collembola. In meadows, however, a species-rich community of forbs and grasses [102] as well as the accumulation of biomass as a result of mulching [96] in combination with the loose mineral planting substrate may have positively influenced Collembola populations. Mowing did not have a strong effect on Collembola, as low and high numbers were found in both mown and unmown meadow spots. This finding can be explained by the close relationship of Collembola to soil, which also reduced the positive responses to vegetation height in other studies on urban green space invertebrates [16].

#### Orthoptera

Orthoptera, mainly grasshoppers (Caelifera) and katydids (Ensifera), were relatively rarely found in both types of green space plots. While Orthoptera can reach high densities and considerable biomass in many grasslands [103, 104], they react sensitively to the intensity of grassland use [58], and can occur in only small numbers in urban contexts [16]. We found that Orthoptera, especially Caelifera, benefited from meadows compared to woody roadside vegetation, as no Caelifera were found in the woody vegetation, but high densities were found in some meadow plots. Apart from the vegetation type, no other factor investigated seemed to influence the numbers of Orthoptera in our study. Although habitat size and isolation can influence Orthoptera abundance and species richness in urban areas [105], we found the highest density of Orthoptera in both study years in one of the smallest plots, a meadow area of only 3.3 m^2^ in the pedestrian zone of a residential area: eight individual Caelifera/m^2^ of suction sample, which is also a high density for extensively mown meadows in ecological compensation areas [46] or natural grasslands [104]. It is possible that the lack of car traffic in the immediate vicinity has reduced the mortality rate of these mobile insects, which may otherwise suffer marked road deaths [31, 106]. High Orthoptera densities in some of our study plots support the idea that even small urban green spaces can be of value to wildlife if basic habitat requirements of species are taken into account [80]. Our study also suggests that pitfall traps provide results biased toward Caelifera, whereas suction sampling provides a more realistic picture of both Caelifera and epigaeic Ensifera (not necessarily crickets). It can therefore be considered as a standardized sampling technique that can provide more comparable data on Orthoptera assemblages for different habitats and differing vegetation heights [107]. Mowing is known to strongly affect Orthoptera [51, 58], but in our study we found no differences between mown and unmown meadow spots. One explanation for this finding could be that unmown spots were small and the highly mobile Orthoptera easily moved from shelters in the unmown area (reducing Orthoptera number in unmown spots) to the mown area, where bare soil and plant regrowth may provide attractive environmental conditions [108].

#### Aphidoidea

Aphidoidea (aphids) were very abundant in pitfall traps but less abundant in suction samples. Aphids were generally more abundant in meadow samples than in samples of woody vegetation. While the highest activity abundance was found in young meadows, the highest densities within the vegetation were found in old meadows. High numbers of aphids in young meadows may be related to easy trapping of aphids that leave small host plants due to plant overexploitation or disturbance [109, 110]. In old meadows, aphids may more rarely reach the ground if disturbed and are thus less likely assessed by pitfall traps. The low number of aphids in woody vegetation is probably related to (1) shrub species identity, with very few aphid species being related to the exotic shrubs studied, and (2) the season, as many aphid species show a host change between primary woody and secondary herbaceous host plants [111, 112]. As a result, aphid density on woody plants is generally higher in spring before dispersal to secondary herbaceous hosts and possibly also in autumn after returning to primary woody hosts. In summer, when sampling took place, many aphid species had switched to non-woody secondary host plants, which may explain the low number of aphids detected in woody vegetation. The finding that mowing had no demonstrable effect on aphid density possibly can be related to host use by aphids: they do not hide but usually occur where they feed. As fresh leaves and shoots, which aphids usually require, do not occur frequently in unmown, dry midsummer meadows, the aphids were not attracted more strongly to unmown areas than to mown meadow spots. In cases where (re)growing plants are available independently of mowing, higher mowing frequencies may reduce aphid numbers [113].

#### Auchenorrhyncha

Auchenorrhyncha (plant- and leafhoppers) occurred in the majority of study plots in both pitfall and suction samples. Auchenorrhyncha activity abundance and density were significantly higher in meadow plots than in woody vegetation, which clearly shows the positive effect of vegetation conversion for this insect taxon. Auchenorrhyncha also reacted sensitively to meadow age: while activity abundance in old meadows was relatively very high, young meadows showed much lower activity abundances in year 1, comparable to the low numbers in woody vegetation. As it can be assumed that pitfall traps catch insects more easily under the open conditions of the young meadow without plant parts growing above the trap, the strong difference between young and old meadows reflects the low numbers of Auchenorrhyncha in this habitat type. In year 2, when plants had completely covered the area of the young meadows, Auchenorrhyncha density in young and old meadows was no longer distinguishable, indicating a rapid population increase of at least some Auchenorrhyncha species in the newly created habitat (preliminary species identification revealed 39 species for old meadows, 23 species for young meadows and 13 species for woody vegetation in year 2; [114]). As already shown by studies in extensively managed meadows [89] and grasslands differing in land-use intensity [115], delayed mowing has greatly increased Auchenorrhyncha density, with densities 94% higher in the unmown than in the mown meadow spots. Although we have found that urban green spaces and especially unmown meadow spots in old meadows were habitat to a considerable number of Auchenorrhyncha, these numbers are much lower than those of optimal rural Auchenorrhyncha habitats, which can reach several 1000 individuals/m^2^ in suitable habitats [116]. Our finding that plot size and distance to the city boundary have not influenced Auchenorrhyncha numbers is consistent with other studies showing that many Auchenorrhyncha species can persist in small habitat patches [117], but it seems nevertheless possible that larger areas of meadow vegetation are needed to ensure optimal habitat heterogeneity [118], especially for populations of some specialized Auchenorrhyncha species [119]. As shown by the differences in Auchenorrhyncha density between young meadows in the year of establishment and one year later–and meadows five years and older, it seems plausible to consider urban meadows to develop growing Auchenorrhyncha populations over time. In this case more and more species may reach the plots and develop populations corresponding to habitat size, mortality factors including car traffic and host plant availability. The Auchenorrhyncha communities thus clearly document changes in habitat quality [120].

#### Heteroptera

Heteroptera (true bugs) occurred in all meadow and most woody vegetation plots. Like other taxa, Heteroptera profited greatly from the conversion to meadow vegetation, as the numbers in old and young meadows were much higher than in woody vegetation. In contrast to the other investigated hemipteran groups (Aphidoidea, Auchnorrhyncha), Heteroptera are not exclusively phytophagous but include different feeding guilds such as zoophagous, zoophytophagous and phytophagous species [121, 122]. Different food sources may allow generalist species to use both established (old) and establishing (young) meadows, which may explain the finding that Heteroptera occurred in equal numbers in young and old meadows in year 1, as opposed to the other hemipterans that occurred in higher numbers either in old or in young meadows. As in other phytophagous taxa, low numbers of Heteroptera in the woody roadside vegetation could be explained by a low number of phytophagous heteropterans feeding on the exotic plants [68] and a low number of other phytophagous organisms serving as food for predatory heteropterans. Interestingly, in the second year after establishment, young meadows seemed to provide a particularly suitable habitat for heteropterans, as the heteropteran density was remarkably high in this vegetation type. It is possible that a high flower supply of short-lived biennial and perennial plants producing flowers in the second year has provided food for flower-, fruit- and seed-feeding species [123–125], and attracted prey for predatory species feeding on other flower visitors. Delayed mowing also increased Heteroptera density compared to mown meadow spots, which is consistent with other studies showing negative effects of mowing on Heteroptera occurrence [126, 127].

#### Coleoptera

Coleoptera (beetles) appeared in almost all plots (only one woody vegetation plot in year 2 was without beetles). The regular occurrence of beetles is also reported from other urban areas [16, 81]. Coleoptera showed a much higher density in meadow plots in year 2, whereas in year 1 no differences in activity abundance between vegetation types were observed. As beetles are particularly diverse in terms of species, but also in terms of life history, this finding of strongly diverging effects of vegetation type depending on the sampling method can probably only be interpreted by a more detailed investigation of the reactions of different beetle groups, which goes beyond the scope of this paper. Beetles represent all major insect feeding guilds [121], and depending on the feeding guild they may prefer different habitat types. Although phytophagous beetles can use both woody and herbaceous plants as food, and trees can host very high beetle densities [128, 129], our study considered exotic shrubs that may host lower numbers of insects than native trees [67, 68, 130]. The meadows, on the other hand, were rich in native plant species that may have favored many phytophagous beetle species. The most commonly sampled beetles in pitfall traps, including carabids and staphylinids, are predominantly carnivorous and less dependent on the presence of certain host plants than phytophagous beetles. Unmown meadow spots led to a greatly increased beetle density, which may be associated with the availability of additional resources such as ripening fruits and seeds, but also possibly with increased shelter. Purely increased spatial complexity of the vegetation should not be a main reason for higher beetle numbers in unmown meadows, as the more complex woody vegetation contained significantly fewer beetles than even mown meadows. Interestingly, Coleoptera together with Nematocera were the only taxa that showed increasing numbers with increasing distance to the city boundary. Contrary to the expectation that rural habitats in the surrounding of cities can serve as a source for insect populations (see [18] for an overview of the relationship between invertebrate numbers in urban and adjacent non-urban areas), this finding rather suggests that the rural environment does not necessarily provide a surplus of immigrating insects. Higher insect numbers at a greater distance from the city boundary may be explained by favorable environmental conditions such as elevated temperatures, but they may also be related to negative influences from the surrounding rural environment, including the drift and transport of pesticides and nitrogen from the surrounding landscape to urban areas by air and water [3, 131].

#### Nematocera

Nematocera (mainly mosquitoes and some midges) were rather rarely found in meadow plots in both years, but were the only taxon to occur in a constantly higher number in woody vegetation. Mowing also reduced the density of the Nematocera. Most Nematocera were mosquitoes (Culicidae), which for decades have been subject to intensive control measures in the Upper Rhine area to which Riedstadt belongs [132]. Strong control measures in the surroundings of the city may also explain our finding of a positive relationship between sampled Nematocera and the distance to the city boundary, a relationship found only for Coleoptera and Nematocera. The lower number of mosquitoes in flower meadows compared to woody vegetation could be explained by the fact that the bushes can serve as shelter for these insects [133]. These shelter effects could also explain the finding that high Nematocera densities were found in one unmown meadow spot. The finding of a reduced mosquito number in meadows compared to the woody roadside vegetation indicates a benefit of the meadows, as they can contribute to reducing nuisance mosquito populations [134]. However, it should be noted that different mosquito species can also react differently to certain environmental parameters [135, 136]. Unmown meadow areas may have a similar effect on mosquito populations as woody vegetation, but this effect seems to be generally smaller, as we have found an increased number of Nematocera in only one unmown meadow spot.

#### Brachycera

Brachycera (flies) were very common in suction samples, but not in pitfall traps, although they were sampled from most plots using both methods. In pitfall traps, the highest activity abundance of Brachycera was found for young meadows, which may be related to the easy accessibility of traps in bare soil, but may also reflect a higher activity of Brachycera sunbathing on the ground or foraging for food–including dog excrements. In year 2, Brachycera densities were generally higher in meadows than woody vegetation, with highest numbers again in young meadows. Since full vegetation cover was reached by young meadows in year 2, other reasons than in year 1 may apply to explain the high numbers of Brachycera in young meadows. Besides a saprophagous diet many flies feed on flowers or other insects [137–139]. The higher availability of these food resources may be reflected in the higher number of Brachycera in flower meadows compared to the woody vegetation, where mainly shelters and sites for sun basking are available. However, a higher structural complexity in addition to the availability of flowers may also favor some Brachycera [140], which is probably reflected in a higher (though not significantly) density in unmown compared to mown meadow plots.

#### Apocrita

Apocrita–here relating to parasitic and aculeate wasps, but not to bees (not sampled) and ants (discussed below)–appeared like Brachycera in most plots and were particularly common in suction samples. In year 1, activity abundance of Apocrita in meadows was 30% lower than in woody vegetation. This could indicate that wasps in woody vegetation foraged more at ground level than in meadows, where more food could be provided by the upper flowering vegetation layers (old meadows), or whose general attractiveness for wasps was low (young meadows). In suction samples, young meadows had the highest Apocrita densities, which may be related to a particularly suitable flower supply in these meadows at the time of collection. The possible positive role of the availability of flower resources for the density of Apocrita is supported by the observation that the densities of other taxa that are typically flower-visitors (Brachycera) or inflorescence consumers (Heteroptera) were also highest in young meadows in year 2. In addition to floral resources, many wasp species are attracted by prey or host organisms [141–143], which, as our study shows, can occur more frequently on flowering meadows than in exotic woody plants. Interestingly, there was no difference between mown and unmown meadow spots. This suggests that either food availability for wasps is not strongly influenced by changes in vegetation structure related to mowing four weeks prior to arthropod assessment, or that the different environmental requirements of this very species-rich taxon [144] can stabilize resource use by wasps in different meadow types [145].

#### Formicidae

Formicidae (ants) are generally abundant in most temperate and tropical terrestrial ecosystems, including urban green spaces [83, 146], and we found the same in our study plots. Formicidae occurred in all plots, in most pitfall samples and in almost all meadow suction samples. Occurrence and density in woody vegetation were lower than in meadow plots, which may be due to climatic reasons, as many opportunistic ants occur in higher densities in open, warmer habitats [147, 148]. Young meadows in year 1 were the most open, warmest habitat, but had (though not significantly) lower activity abundance than old meadows. This indicates that not only climatic variables, but also the time for the establishment and recovery of ant communities after the creation of the meadows [71] and the availability of food resources could be important determinants of ant numbers. As far as food resources are concerned, exotic woody vegetation probably provides lower food quantities, since fewer insects use these plants as hosts [149, 150], although some insect species may thrive on such plants and can thus provide food for opportunistic ant species [22, 151]. In the case of pitfall traps, ants in woody vegetation may tend to use higher vegetation layers for foraging, which impairs the effectiveness of pitfall traps. As ant density was also lower in suction samples of woody vegetation, the generally lower numbers in woody vegetation appear not to be related to foraging area, but reflect densities in habitats that differ in vegetation density. The assumption of a negative influence of vegetation density is supported by our observation that ant densities in mown meadows were 10% higher than in unmown meadows despite the larger vegetation volume and the resulting higher complexity, which is known to positively affect ant communities [147, 152]. Missing effects of patch size support results of other studies on ants in urban green spaces, which also did not find clear indications of the influence of green space size on patch occupancy by ants [153].

#### Total arthropods

Considering the total arthropod communities of the investigated plots, a general positive effect of the conversion of woody vegetation into wildflower meadows on arthropod activity abundance (increase 212%) and density (increase 260%) was observed. This finding clearly shows that the conversion of exotic roadside vegetation into wildflower meadows can contribute to the establishment of higher arthropod numbers in urban areas. Since many insect and arthropod populations in rural areas are currently threatened by high land use pressures [3, 62], and urban areas are constantly increasing in size [154, 155], measures to improve the quality of urban green spaces for arthropods can be seen as an increasingly important contribution to arthropod conservation. In addition to the direct conservation of arthropods, these measures can also protect and promote other taxa, including many insectivorous vertebrates [81, 156], and ecosystem services such as pollination, pest control and nutrient cycling [26, 157]. Besides transforming unsuitable habitats into flower meadows [27], maintenance measures, including mowing, can be directed towards the goal of arthropod conservation [50, 158]. Looking at the total arthropod communities, arthropod responses to delayed mowing were not uniform, but overall this measure seemed to be favorable to support arthropods, leading to a general increase in arthropod density of 63%.

### Costs of green space maintenance

Our analyses of the costs of maintenance of urban green space revealed that maintenance costs of flower meadows can be considerably lower than those of other green space types such as woody roadside plantings. In the original woody condition, the workers had to cut the bushes twice a year. The green areas vary greatly in size and shape. In addition, they are located in residential areas with small streets and parking spaces and are therefore difficult to access. Most of the work had to be done manually with hedge trimmers and the material had to be transported to the composting plant. The new meadows are cut with mowers and brush cutters, which makes the work much easier and faster–and only the first growth is collected and transported to the composting plant. Although our cost estimates are therefore not directly transferable to other communities, the cost differences between the vegetation types we studied are consistent with other sources of information, which indicate–for roadside vegetation–considerably lower costs for the maintenance of "landscape lawns" than for frequently mown "utility lawns" (three times more expensive than landscape lawns) or woody areas (six times more expensive) [159]. Therefore, our cost estimates confirmed that wildflower meadows can be a promising option not only to increase the value of green spaces for biodiversity [18], but also to reduce maintenance costs [26, 48, 160]. This fact may lead to an increased planting of urban flower meadows in the future, since in addition to positive effects on biodiversity and context-dependent considerations of aesthetics and public perception, human resources and economic sustainability are important [48]. Our investigations have not shown marked positive relationships between arthropod densities and increasing green space size, therefore the total number of arthropods supported by a green space most likely increases rather linearly with total area. Nevertheless, considering the higher economic efficiency and the expected higher number of species in larger green areas [76] it seems advisable to create larger green spaces if possible.

## Conclusion

Considering the decline of insects and other terrestrial arthropods reported for various regions in central Europe and worldwide [1–6, 15, 161, 162], and the need to better understand the extent and the drivers of decline [163, 164], our study demonstrates the potential of urban wildflower meadows to support various arthropod taxa in urban areas. In a world of increasing urbanization [80, 155, 165], the greatly increased density of a variety of arthropods in wildflower meadows compared to exotic shrubs represents an enhanced value of appropriately managed urban green spaces for biodiversity conservation. In addition to providing valuable habitat for different arthropod taxa, urban wildflower meadows have proven to be very cost-effective, which can lead to a win-win situation with increased habitat value and lower maintenance costs.

## Supporting information

S1 Table Overview on taxa that were considered for sorting of arthropods sampled by pitfall traps and suction sampling. Taxa are ordered according to number of individuals assigned to each taxon.
(PDF)

S2 Table “Riedstadt seed mixtures” for inner urban green spaces, City of Riedstadt, Matthias Harnisch 2009. Red: Species / varieties that were not found in a 2012/13 monitoring: Hilmer, M. (2013): Vegetation in der Stadt—Aussaat von Magerrasen in Riedstadt“, Masterthesis, University of Gießen, Germany.
(PDF)

S3 Table Comparison of arthropod numbers collected by pitfall traps in the center or at the edge of study plots in year 1. Arthropod numbers were summed up across five sampling days for each plot and trap location and compared by paired samples Wilcoxon tests (N = 40). In case of single missing traps, both traps were omitted from analysis.
(PDF)

S4 Table Comparison of arthropod density assessed by suction sampling in mown and unmown meadow spots in year 2. Arthropod densities were compared for the subset of meadow plots that contained both mown and unmown spots by paired t-tests (test statistic: t) or paired samples Wilcoxon tests (test statistic: W); ns = not significant. See Fig 4 and Table 2 for details on arthropod numbers.
(PDF)

S1 File Data set underlying the reported study. YEAR: Year of sampling; 2015 or 2016. TYPE: Type of green space, three categories; Old = wildflower plots converted in February 2010, Young = wildflower plots converted in March 2015, Woody = plots with original woody vegetation. PLOT: Plot number; P1—P43. DISTRICT: Areas of Riedstadt encompassing plots with high similarity regarding the characteristics of the adjacent rural area, five categories. DISTANCE: Linear distance to nearest rural (non-built-up) area (m). AREA: Size of green space plots (m^2^). SAMPLE: Sampling event 1 to 5 in 2015 (between 9 June and 16 July). POSITION: Pitfall trap position in the plot; C = center, E = edge. Arthropod taxa: Individual numbers. Total.Arthropods: Total number of arthropod individuals sampled and sorted during the study.
(TXT)
