# Supplementary material for: Flower power in the city: Replacing roadside shrubs by wildflower meadows increases insect numbers and reduces maintenance costs
Source: PLoS One. 2020 Jun 9;15(6):e0234327. doi: 10.1371/journal.pone.0234327 (PMC7282654; doi:10.1371/journal.pone.0234327)
Supplement: S1 Table — Taxa are ordered according to number of individuals assigned to each taxon. (PDF) [file pone.0234327.s001.pdf]

S1 Table. Overview on taxa that were considered for sorting of arthropods sampled by pitfall traps and suction sampling. Taxa are ordered according to number of individuals assigned to each taxon.

**Taxon**	**Individual number**	**Percent of total**
**Collembola**	8452	31.0
**Formicidae**	6347	23.3
**Aphidoidea**	2275	8.4
**Heteroptera**	1791	6.6
**Auchenorrhyncha**	1658	6.1
**Coleoptera**	1594	5.9
**Araneae**	1529	5.6
**Brachycera**	1263	4.6
**Apocrita**	655	2.4
**Isopoda**	412	1.5
Insect larvae	372	1.4
Acari	241	0.9
**Nematocera**	176	0.6
**Opiliones**	144	0.5
**Orthoptera**	98	0.4
Blattodea	76	0.3
Dermaptera	61	0.2
Lepidoptera	48	0.2
Myriopoda	28	0.1
Neuroptera	19	0.1
