# Supplementary material for: Flower power in the city: Replacing roadside shrubs by wildflower meadows increases insect numbers and reduces maintenance costs
Source: PLoS One. 2020 Jun 9;15(6):e0234327. doi: 10.1371/journal.pone.0234327 (PMC7282654; doi:10.1371/journal.pone.0234327)
Supplement: S2 Table — Red: Species / varieties that were not found in a 2012/13 monitoring: Hilmer, M. (2013): Vegetation in der Stadt—Aussaat von Magerrasen in Riedstadt“, Masterthesis, University of Gießen, Germany. (PDF) [file pone.0234327.s002.pdf]

S2 Table. “Riedstadt seed mixtures” for inner urban green spaces, City of Riedstadt, Matthias Harnisch 2009. Red: Species / varieties that were not found in a 2012/13 monitoring: Hilmer, M. (2013): Vegetation in der Stadt - Aussaat von Magerrasen in Riedstadt“, Masterthesis, University of Gießen, Germany.

<table>
<tr>
<td rowspan="2">Basic seed mixture

Agrimonia eupatoria
Anchusa officinalis
Artemisia campestris
Berteroa incana
Carduus nutans
Centaurea cyanus
Centaurea scabiosa
Centaurea stoebe
Cichorium intybus
Clinopodium vulgare
Daucus carota
Dianthus carthusianorum
Dianthus superbus
Echium vulgare
Eryngium campestre
Falcaria vulgaris
Galium verum
Geranium sanguineum
Knautia arvensis
Leucanthemum vulgare
Linaria vulgaris
Linum perenne
Malva moschata
Oenothera biennis
Origanum vulgare
Papaver dubium
Papaver rhoeas
Pimpinella saxifraga
Plantago lanceolata
Salvia pratensis
Tragopogon dubium
Vaccaria hispanica
Verbascum nigrum
Verbena officinalis
Veronica teucrium</td>
<td>Seed mixture for small areas

Armeria maritima
Artemisia campestris
Centaurea stoebe
Corynephorus canescens
Euphorbia cyparissias
Helianthemum nummularium
Koeleria pyramidata
Helichrysum arenarium
Origanum vulgare
Saponaria ocymoides
Sedum acre
Silene otites
Stipa capillata
Thymus pulegioides
Viola arvensis</td>
<td>Additional seed mixture for larger areas

Echinops sphaerocephalus
Epilobium angustifolium
Malva alcea
Pastinaca sativa
Reseda luteola
Verbascum densiflorum</td>
</tr>
<tr>
<td>Seed mixture to promote the conversion of grass-dominated lawns into meadows

Agrimonia eupatoria
Centaurea jacea
Clinopodium vulgare
Daucus carota
Galium verum
Geranium sanguineum
Knautia arvensis
Leucanthemum vulgare
Linaria vulgaris
Pimpinella saxifraga
Plantago lanceolata
Rhinanthus alectorolophus
Salvia pratensis
Tragopogon dubium
Veronica teucrium</td>
<td>Planted herbaceous perennials as basic structure

Aster novi-angliae
Calamagrostis x acutiflora "Karl Förster"
Molinia arundinacea "Karl Förster“
Muscari neglectum
Narcissus poeticus “Actea”
Narcissus pseudonarcissus
Rudbeckia fulgida var. deamii
Sedum telephium –Hybrids
Tulipa sylvestris</td>
</tr>
</table>
