# Supplementary material for: Flower power in the city: Replacing roadside shrubs by wildflower meadows increases insect numbers and reduces maintenance costs
Source: PLoS One. 2020 Jun 9;15(6):e0234327. doi: 10.1371/journal.pone.0234327 (PMC7282654; doi:10.1371/journal.pone.0234327)
Supplement: S3 Table — Arthropod numbers were summed up across five sampling days for each plot and trap location and compared by paired samples Wilcoxon tests (N = 40). In case of single missing traps, both traps were omitted from analysis. (PDF) [file pone.0234327.s003.pdf]

S3 Table. Comparison of arthropod numbers collected by pitfall traps in the center or at the edge of study plots in year 1. Arthropod numbers were summed up across five sampling days for each plot and trap location and compared by paired samples Wilcoxon tests (N = 40). In case of single missing traps, both traps were omitted from analysis.

**Taxon**	**Median arthropod number center**	**Median arthropod number edge**	**V**	***P***
Opiliones	1	0	70.5	0.915
Araneae	6	5	405	0.840
Isopoda	1	1	221.5	0.677
Collembola	36	35	405.5	0.834
Orthoptera	0	0	71.5	0.239
Aphidoidea	7.5	5.5	392	0.761
Auchenorrhyncha	4	4.5	327.5	0.406
Heteroptera	3	3	348	0.230
Coleoptera	3	4	333	0.547
Nematocera	0.5	1	350	0.103
Brachycera	1.5	2	227	0.589
Apocrita	1	1	265.5	0.986
Formicidae	40.5	44	456	0.362
Total arthropods	166	139	508	0.192
