# Supplementary material for: Flower power in the city: Replacing roadside shrubs by wildflower meadows increases insect numbers and reduces maintenance costs
Source: PLoS One. 2020 Jun 9;15(6):e0234327. doi: 10.1371/journal.pone.0234327 (PMC7282654; doi:10.1371/journal.pone.0234327)
Supplement: S4 Table — Arthropod densities were compared for the subset of meadow plots that contained both mown and unmown spots by paired t-tests (test statistic: t) or paired samples Wilcoxon tests (test statistic: W); ns = not significant. See Fig 4 and Table 2 for details on arthropod numbers. (PDF) [file pone.0234327.s004.pdf]

S4 Table. Comparison of arthropod density assessed by suction sampling in mown and unmown meadow spots in year 2. Arthropod densities were compared for the subset of meadow plots that contained both mown and unmown spots by paired t-tests (test statistic: t) or paired samples Wilcoxon tests (test statistic: W); ns = not significant. See Fig 4 and Table 2 for details on arthropod numbers.

**Taxon**	**Test statistic**	***P***
Opiliones	W = 7.00	0.450
Araneae	t = 1.77	0.115
Isopoda	W = 10	0.068
Collembola	W = 16	0.735
Orthoptera	W = 14	0.462
Aphidoidea	W = 26	0.262
Auchenorrhyncha	W = 44	**0.011**
Heteroptera	t = 2.48	**0.038**
Coleoptera	t = 3.88	**0.005**
Nematocera	W = 4	0.593
Brachycera	W = 36	0.110
Apocrita	t = 0.18	0.863
Formicidae	W = 23	0.953
Total arthropods	t = 3.21	**0.012**
